# Perception, Practices, and Barriers to Cervical Cancer Screening Among Women in Rural and Urban Slums of Eastern India: A Community-Based Cross-Sectional Study

**DOI:** 10.7759/cureus.102449

**Published:** 2026-01-27

**Authors:** Geeta C Acharya, Sumelika Das, Avnika Jasuja, Ipsita Debata

**Affiliations:** 1 Department of Community Medicine, Kalinga Institute of Medical Sciences, Bhubaneswar, IND; 2 Department of Pathology, Andaman and Nicobar Islands Institute of Medical Sciences, Sri Vijaya Puram, IND

**Keywords:** awareness, barriers, cervical cancer, india, rural women, screening, urban slums

## Abstract

Background

Despite being avoidable with prompt screening, one of the notable causes of death among Indian women is still cervical cancer. Awareness, access to screening, and perceptions of risk differ considerably, especially in rural and underserved urban populations.

Objective

The purpose of the study was to assess awareness, perceptions, screening practices, and perceived barriers related to screening of cervical cancer among women, between the ages of 30 and 65 years, living in rural and urban slum areas of Eastern India.

Methods

A descriptive cross-sectional study was carried out between November 2024 and February 2025 in the community under the field practice areas of a tertiary care hospital in Bhubaneswar, Odisha. A total of 400 women (200 each from rural and urban slums) were chosen by a two-stage random sampling method. Information was gathered using a predesigned, pre-tested semi-structured questionnaire. The data was analyzed using descriptive statistics and binary logistic regression. Statistical significance was defined as a p-value of less than 0.05.

Results

The participants' average age was 39.09 ± 8.93 years. Only 27 (6.7%) women had awareness regarding screening of cervical cancer and had ever undergone screening, all of which occurred in private health care facilities. Awareness regarding free government-run screening services was poor, with 192/200 (96.0%) of urban and 189/200 (94.5%) of rural women being unaware of such services. Major barriers to screening included mistrust in public health services (31.0%), difficulty accessing health centers (31.8%), and fear of pain (11.8%). Mistrust in health services and accessibility issues were significantly associated with non-utilization of screening services (p < 0.001). Among women who had undergone screening, healthcare workers were the primary source of motivation.

Conclusion

Less than one in fifteen women in rural and urban slum regions had ever been screened for cervical cancer, and there were significant gaps in knowledge of government-provided programs. Perceptual and structural obstacles, such as mistrust and limited accessibility, were prevalent. To increase screening participation, community-based awareness must be strengthened, public health services must be trusted, and accessibility must be improved.

## Introduction

One of the rare cancers that can be mostly avoided with prompt screening, early detection, and appropriate treatment is cervical cancer. It continues to be a major source of women’s mortality and morbidity globally, particularly in low- and middle-income countries (LMICs), despite the availability of efficient preventive strategies. Globally, approximately 604,000 women were affected with cervical cancer, and more than 342,000 fatalities were reported in 2020 alone [1]. The burden is particularly disproportionate in India, which is responsible for over 25% of cervical cancer fatalities worldwide, with over 120,000 new cases and approximately 77,000 deaths annually [2].

Recognizing the preventable aspect of cervical cancer, public health initiatives in India, including the National Cancer Control Programme and the more recent National Programme for Prevention and Control of Cancer, Diabetes, Cardiovascular Diseases and Stroke (NPCDCS), have emphasized early screening using Visual Inspection with Acetic Acid (VIA) and Papanicolaou (Pap) smear testing, especially in primary healthcare level [3]. The World Health Organization advises routine cervical cancer screening starting at age 30 because, particularly in low-resource settings, the risk of persistent human papillomavirus infection and development to precancerous lesions increases significantly after this age [4]. Until the age of 65, screening is typically recommended; after that, the value diminishes for women who have undergone sufficient prior screening. The 30- to 65-year-old age group is the most suitable target demographic for cervical cancer screening, according to evidence from India, which balances screening yield, cost-effectiveness, and invasive disease prevention [5].

Despite this, screening coverage remains unacceptably low. National Family Health Survey-5 data reported that fewer than 2% of women from India, aged between 30 and 49 years, have ever had a cervical screening [3]. Although recent programmatic data suggest an expansion of screening through Ayushman Arogya Mandirs using VIA, with over 100 million women screened by mid-2025, this coverage remains uneven and does not adequately reflect uptake among socioeconomically disadvantaged populations [6].

Multiple barriers contribute to low screening uptake in India, including limited knowledge and awareness, sociocultural stigma, fear of pain or diagnosis, misconceptions regarding screening procedures, and poor accessibility to healthcare facilities. These barriers are particularly pronounced among women residing in rural areas and urban slums [7]. These communities often face the double burden of poverty and poor health infrastructure, leaving a gap between policy intent and ground reality. Consequently, a substantial gap persists between the accessibility and use of cervical cancer screening services at the community level.

In this context, the study was undertaken to assess the awareness, perceptions, and practices related to cervical cancer screening and to determine the perceived barriers to screening among women aged 30 - 65 years residing in rural and urban slum areas of Eastern India.

## Materials and methods

Study design and setting

This cross-sectional study was conducted in the community between November 2024 and February 2025 at selected urban slums and rural villages under the field practice areas of the Urban Health and Training Centre (UHTC) and Rural Health and Training Centre (RHTC), respectively. Both centers run under the Community Medicine Department of a tertiary medical college and teaching hospital in Bhubaneswar, Odisha, India.

Study population and eligibility criteria

The study participants were women between the ages of 30 and 65 who had been residing in the selected areas for a minimum of six months. Women suffering from terminal health conditions and women diagnosed with psychiatric or convulsive disorders were excluded from the study.

Sample size

The sample size was determined using the formula $n = \frac{4pq}{l^2}$, where p represents prevalence, q = 1-p, and l denotes allowable error. Assuming a prevalence of 50% to ensure maximum variability and an allowable error of 5%, the estimated sample size was 400 participants. This included 200 women, each from urban slums and rural villages.

Sampling technique

The study participants were chosen using a two-stage sampling process. In the first stage, five urban slums and five rural villages were chosen by simple random sampling from the list of eligible areas under the UHTC and RHTC, respectively. In the second stage, 40 eligible women were enrolled from each selected slum or village using a spatial random walk method.

The direction of household visits was determined by the bottle-spinning technique from a central point in each area, a field-friendly method that ensured the randomization of entry points. Data collection began at the first household in the indicated direction, and investigators proceeded sequentially to adjacent households. One eligible woman was interviewed per household. Locked houses and non-consenting participants were excluded without replacement. The process continued until the required sample size of 40 women per area was achieved, yielding a total of 400 participants.

Data collection tool and procedure

Data were collected by a predesigned questionnaire (Appendix 1), adapted from published literature and standard national guidelines [3,5,6,7]. To ensure content relevance, clarity, and contextual appropriateness, the questionnaire items were examined by subject matter experts in public health and community medicine. It was devised in English and translated into the local language, Odia, for field use. The questionnaire was back-translated to ensure conceptual and linguistic accuracy. To evaluate feasibility and clarity, a pilot test was carried out in a similar population from other urban slums and rural areas. Data from the pilot test were excluded from the final study, and no modifications were needed after the pilot.

Individual questionnaire items with binary (Yes/No) or categorical response options were used to evaluate knowledge, screening practices, and perceived barriers. No summative or aggregate scores were computed for knowledge, attitudes, or perceived barriers; each item was examined separately. The questionnaire included the following domains: Socio-demographic characteristics included residence, housing type, age, marital status, age at menarche, obstetric history, family history of cancer, menstrual history, education, occupation, and socioeconomic status. Knowledge regarding cervical cancer and screening included questions regarding awareness about cancer, signs and symptoms, the affected age group, the screening program, screening locations, and vaccination. Cervical cancer screening practices included questions on contraceptive use, menstrual hygiene practices, history of cervical cancer screening, type of screening procedure undergone, and source of encouragement or referral. Perceived barriers and attitude towards screening were assessed using questions addressing beliefs and attitudes regarding screening (e.g., fear of pain, embarrassment, stigma, mistrust, accessibility issues, misconceptions). 

Face-to-face interviews were conducted by trained female field investigators and medical interns under faculty supervision to ensure participant comfort, cultural sensitivity, and data reliability.

Statistical analysis

Data were analyzed using IBM SPSS Statistics for Windows, version 21.0 (IBM Corp., Armonk, NY, USA). The results were summarized using descriptive statistics, such as frequencies, percentages, means, and standard deviations. To find independent predictors of cervical cancer screening behavior, binary logistic regression analysis was used. Every variable was considered categorical. Responses were coded as binary variables (Yes = 1, No = 0) for regression analysis. There was no conversion of summed or continuous scores into binary variables. Statistical significance was defined as a p-value of less than 0.05.

Ethical considerations

The Institutional Ethics Committee of Kalinga Institute of Medical Sciences, Bhubaneswar, granted ethical approval (KIIT/KIMS/IEC/2280/2025). Every participant provided written informed consent. Participants were made aware that participation in the study was entirely optional and that their responses would only be used for academic purposes, and privacy and confidentiality were upheld throughout.

## Results

The study included 400 women in total, with equal representation from both urban slum areas and rural villages (200 participants each). The study participants’ average age was 39.09 ± 8.93 years. Table 1 depicts the sociodemographic details of the study participants.

**Table 1:** Socio-demographic characteristics of study participants (n = 400)

Variable	Category	Frequency (%)
Residence	Urban slum	200 (50.0)
	Rural	200 (50.0)
Age group (years)	30–39	168 (42.0)
	40–49	142 (35.5)
	50–65	90 (22.5)
Marital status	Single	63 (15.8)
	Married	325 (81.3)
	Widowed/Divorced	12 (3.0)
Education	Illiterate	96 (24.0)
	Primary	118 (29.5)
	Secondary & above	186 (46.5)

**Figure 1:**
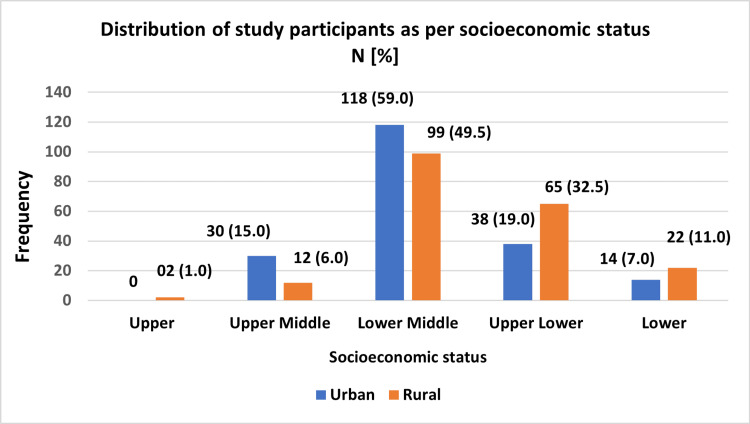
Distribution of study participants according to socioeconomic status as per the Modified BG Prasad Scale (2024) Modified BG Prasad scale (2024) [8].

Based on the Modified BG Prasad Socioeconomic Scale 2024 [8], 139 (34.75%) participants belonged to the lower socioeconomic category, as depicted in Figure 1.

An overview of study participants' knowledge, practices, and perceived barriers for cervical cancer screening is provided in Table 2. All variables are presented as frequencies and percentages after being categorically analyzed. On assessing the knowledge, a substantial proportion of participants, 276 (69%), reported that they had never heard of cervical cancer. Awareness of free government-provided cervical cancer screening services was remarkably low, with 192/200 (96%) of urban slum residents and 189/200 (94.5%) of rural women being unaware of such services, resulting in an overall unawareness rate of 95.25%.

**Table 2:** Knowledge, practices, and perceived barriers related to cervical cancer screening (n = 400) Values are presented as n (%); n = 400

Domain	Item	Yes n (%)	No n (%)
Knowledge	Heard about cervical cancer	124 (31.0)	276 (69.0)
	Aware of the affected age group	51 (12.7)	349 (87.3)
	Aware of the screening program	36 (9)	364 (91)
	Aware of screening locations	53 (13.2)	347 (86.8)
	Aware of human papillomavirus (HPV) vaccination	22 (5.5)	378 (94.5)
	Aware of signs and symptoms	54 (13.5)	346 (86.5)
Practices	Use of contraception	196 (49.0)	204 (51.0)
	Appropriate menstrual hygiene practices	327 (81.8)	73 (18.2)
	Ever undergone cervical cancer screening	14 (3.5)	386 (96.5)
	Encouraged for screening	16 (4.0)	384 (96.0)
Barriers	Screening procedure perceived as painful	47 (11.8)	353 (88.2)
	Lack of family support	29 (7.3)	371 (92.7)
	Difficulty accessing health facilities	127 (31.8)	273 (68.3)
	Belief that disease develops after screening	184 (46.0)	216 (54.0)
	Mistrust in health centers	124 (31.0)	276 (69.0)
	Lack of awareness due to poor promotion	307 (76.8)	93 (23.3)
	Fear of stigma	77 (19.3)	323 (80.8)

Among the screened participants, the majority were younger than 45 years, 16/27 (59.2%), and this age group showed a higher likelihood of screening uptake, although not significant (p = 0.418). For most of these women, the screening was initiated following a recommendation from a healthcare provider, which influenced 16/27 (59.3%) of screened women to undergo the procedure.

Among participants who had never been screened for cervical cancer, several barriers were identified, many of which showed statistical significance with non-utilization of screening services. Difficulty in accessing health centers (χ2 = 7.91, p = 0.004), mistrust in health centers (χ2 = 5.35, p = 0.021), and anticipated out-of-pocket expense (χ² = 0.11, p = 0.018) were found to be significant barriers to cervical cancer screening uptake among women from both rural and urban areas, as depicted in Table 3.

**Table 3:** Knowledge, attitudes, practices, and perceived barriers related to cervical cancer screening and their association with screening uptake (n = 400) Values expressed as a number (%). Statistical test: Chi-square test of independence comparing each variable with cervical cancer screening uptake (ever screened vs never screened); p < 0.05 is considered statistically significant. *Multiple responses permitted for perceived barriers.

Variable	Category		Total n (%)	Ever Screened n (%)	Never Screened n (%)	χ²	p-value
Awareness of cervical cancer	Yes		122 (30.5)	22 (18.0)	100 (82.0)	35.50	< 0.001
	No		278 (69.5)	5 (1.8)	273 (98.2)		
Age group	< 45 years		207 (51.8)	16 (59.2)	191 (51.2)	0.65	0.418
	≥ 45 years		193 (48.2)	11 (40.8)	182 (48.8)		
Perceived barriers to screening*	Perceived screening as painful	Yes	47 (11.8)	1 (3.7)	46 (12.3)	1.80	0.178
		No	353 (88.2)	26 (96.3)	327 (87.7)		
	Difficulty accessing health centers	Yes	127 (31.8)	2 (7.4)	125 (33.5)	7.91	0.004
		No	273 (68.2)	25 (92.6)	248 (66.5)		
	Fear of stigma	Yes	77 (19.3)	2 (7.4)	75 (20.1)	2.61	0.106
		No	323 (23.0)	25 (92.6)	298 (79.9)		
	Mistrust in health centers	Yes	124 (31.0)	3 (11.1)	121 (32.4)	5.35	0.021
		No	276 (69.0)	24 (88.9)	252 (67.6)		
	Anticipated out-of-pocket expense	Yes	113 (28.3)	4 (14.8)	109 (29.2)	0.11	0.018
		No	287 (71.7)	23 (85.2)	264 (70.8)		

As depicted in Table 4, binary logistic regression analysis demonstrated that socio-demographic variables like age, level of education, and socio-economic status showed statistically significant associations with screening knowledge (p = 0.043, p = 0.041, and p = 0.018, respectively). Mistrust in health centers was also significantly associated with both knowledge and favorable attitudes towards screening (p = 0.034, p < 0.001, respectively).

**Table 4:** Association of selected characteristics with knowledge, attitude, and cervical cancer screening practices Reference categories: Higher socioeconomic status; no mistrust in health centers; higher education levels. Statistical test: Binary logistic regression; p < 0.05 is considered statistically significant. Abbreviations: OR, odds ratio; CI, confidence interval.

Characteristic	Knowledge OR (95% CI)	p-value	Favourable Attitude OR (95% CI)	p-value	Practices (Ever Screened) OR (95% CI)	p-value
Age	0.95 (0.91–1.00)	0.043	1.00 (0.98–1.03)	0.892	0.99 (0.95–1.04)	0.744
Education	1.34 (1.01–1.76)	0.041	0.96 (0.82–1.12)	0.600	0.80 (0.60–1.05)	0.112
Socioeconomic status	0.57 (0.36–0.91)	0.018	0.82 (0.61–1.09)	0.176	0.67 (0.40–1.15)	0.148
Mistrust in health centres	0.36 (0.14–0.93)	0.034	10.13 (5.94–17.25)	<0.001	0.72 (0.29–1.79)	0.480
Fear of stigma	2.71 (1.21–6.08)	0.015	1.19 (0.66–2.13)	0.560	1.08 (0.39–3.01)	0.880

Overall, the study reported low uptake of cervical cancer screening, poor awareness of available services, and the presence of deeply rooted practical and psychosocial barriers that hinder women from accessing potentially life-saving services. Gaps in communication, trust, and service delivery within the public health system are further highlighted by screened women's exclusive use of private healthcare facilities. To boost screening adoption, community-level knowledge must be strengthened, public health services must be trusted, and screening programs must be made more accessible and of higher quality.

## Discussion

The present study emphasizes the persistently poor uptake of screening for cervical cancer in India, particularly among socioeconomically disadvantaged women, underscoring a substantial public health gap. The observed screening prevalence of 6.7% is alarmingly low and aligns with findings reported by Srivastava et al. (2022), who reported a prevalence of 3.9% among women in Delhi and Haryana [9]. Collectively, these findings suggest that despite national recommendations and the inclusion of screening for cervical cancer offered by the National Programme for Prevention and Control of Cancer, Diabetes, Cardiovascular Diseases, and Stroke (NPCDCS), population-level screening coverage remains far from adequate.

A particularly concerning finding in the present study is that all women who had undergone screening accessed services exclusively through private healthcare facilities, with no reported utilization of government screening services. This reflects a substantial gap in awareness regarding the availability of free cervical cancer screening in public health centers, as well as a lack of trust in government healthcare services. Similar patterns have been reported in other Indian studies, where perceived poor quality of care, breaches in privacy concerns, and negative attitude of healthcare providers have been identified as deterrents to the use of public-sector screening services.

The barriers identified in this study, including financial concerns, fear, stigma, distance to health facilities, and lack of information, are consistent with findings from multiple Indian settings. Studies by Ramaiah and Jayarama (2018) [10] and Naik et al. (2017) [11] reported poor awareness and sociocultural resistance to gynaecological examinations in rural Karnataka and urban Maharashtra, respectively. Olubodun et al. (2019) [12] further emphasized that fear and embarrassment acted as major deterrents to screening uptake. In Gujarat, Murugan et al. (2024) [13] reported that fewer than 10% of rural women had ever been screened, citing stigma, fatalistic beliefs, gender inequities, and low prioritization of women’s health as key barriers. These findings suggest that low screening uptake is influenced not only by service availability but also by sociocultural norms and deficits in trust within the health system.

International evidence reflects similar challenges across low- and middle-income countries. Studies from Ghana [14], Zimbabwe [15], and Iran [16] have consistently identified cultural taboos, misinformation, and fear of diagnosis as key predictors of non-screening behaviour, indicating that barriers to cervical cancer screening share common structural and sociocultural determinants across diverse settings.

An important finding of the present study was that health worker contact emerged as a significant motivating factor for screening uptake. Evidence from India [17,18] and Botswana [19] similarly demonstrates that interpersonal communication and community-based education delivered by frontline health workers can improve awareness and acceptance of screening for cervical cancer. This emphasizes the potential role that Accredited Social Health Activists (ASHAs), Auxiliary Nurse Midwives (ANMs), and other community health workers can play in bolstering screening programs via focused teaching and trust-building measures.

Socioeconomic factors, including low levels of education, limited income, and unemployment, were also linked to poor screening uptake in the present study. These findings were consistent with earlier studies by Lin (2008) [20], Wu (2003) [21], and Ba et al. (2021) [22], which demonstrated strong associations between socioeconomic disadvantage and reduced utilization of preventive health services.

Emerging strategies such as HPV self-sampling kits and mobile outreach services may help address several of the identified barriers. Evidence from India and other LMICs suggests that self-sampling approaches, as proposed by Bhatia et al. (2021) [23], may be particularly acceptable to women who are hesitant to undergo facility-based pelvic examinations. Integrating such approaches with strengthened community engagement and improved public health infrastructure and delivery could enhance cervical cancer screening coverage in underserved populations.

Recommendations

The results of the study indicate that increasing community-level knowledge through culturally relevant health education given in local languages is necessary to improve cervical cancer screening uptake in low-resource settings. Frontline healthcare professionals, such as Anganwadi workers and Accredited Social Health Activists (ASHAs), can be crucial in educating the public, dispelling myths, and encouraging women to use screening programs. It may be possible to increase awareness and normalize preventative gynecological care by incorporating cervical cancer screening messages into already-existing maternity and reproductive health platforms, such as Village Health and Nutrition Days.

It is equally necessary to address psychological and structural impediments. While consistent service delivery and better communication regarding free screening services may lessen mistrust in public health facilities, strategies like the introduction of human papillomavirus self-sampling approaches may help overcome embarrassment and reluctance toward facility-based screening. In an effort to increase awareness, trust, and screening uptake, community health workers participated in targeted health education sessions after the study's findings were shared, and talks with local health authorities were started to organize recurring screening camps and improve referral connections.

Limitations

As a cross-sectional study, the information was captured at a single point in time, and therefore, does not allow causal inferences between knowledge, attitudes, and cervical cancer screening practices. Recall bias or social desirability bias could have also arisen from the use of self-reported data, especially for sensitive issues related to reproductive health. However, the use of a representative sample and a comparative urban-rural approach strengthened the relevance of our findings. Additionally, interviews conducted in participants’ homes may have influenced responses in joint or extended family settings. The study did not include male partners or family decision-makers, whose influence may affect women’s healthcare-seeking behaviour; this warrants future qualitative research.

## Conclusions

This community-based study demonstrates that screening uptake for cervical cancer among women in urban slums and rural areas of Eastern India remains low. Fewer than one in fifteen women had ever undergone cervical cancer screening, and nearly three-fourths of participants lacked awareness regarding the availability of free screening services at government health facilities. Structural and psychosocial barriers, including mistrust in public health services, limited accessibility, and concerns related to stigma or embarrassment, were commonly reported. Despite the availability of cost-free screening under national programs, utilization was limited to private healthcare facilities, underscoring gaps in public health communication and trust. Strengthening community-level awareness through frontline health workers, improving accessibility, and enhancing trust in public sector services are essential to enhance screening uptake and to support progress toward cervical cancer prevention goals.

## Appendices

Appendix 1: study questionnaire

**Table 5:** Study questionnaire Credits: Geeta Chand Acharya, Sumelika Das, Avnika Jasuja, Ipsita Debata

Section	Item / Question (English)	Item / Question (Odia)	Response Options (English)	Response Options (Odia)
I. Socio-demographic details	Residence	ବସୋବାସ ସ୍ଥାନ	Urban slum / Rural	ସହରୀ ବସ୍ତି / ଗ୍ରାମୀଣ
	Type of house	ଘରର ପ୍ରକାର	Pucca / Semi-pucca / Kuccha	ପକ୍କା / ଅର୍ଧପକ୍କା / କଚ୍ଚା
	Age (years)	ବୟସ (ବର୍ଷ)		
	Marital status	ବିବାହ ସ୍ଥିତି	Married / Unmarried / Widowed / Divorced	ବିବାହିତ / ଅବିବାହିତ / ବିଧବା / ବିଚ୍ଛିନ୍ନ
	Age at menarche (years)	ପ୍ରଥମ ରଜସ୍ରାବ ବୟସ (ବର୍ଷ)		
	Obstetric history	ଗର୍ଭାବସ୍ଥା ସମ୍ବନ୍ଧୀୟ ଇତିହାସ	Gravida, Para, Abortions, Living children	ଗର୍ଭ ସଂଖ୍ୟା, ପ୍ରସବ, ଗର୍ଭପାତ, ବଞ୍ଚିତ ସନ୍ତାନ
	Family history of cancer	ପରିବାରରେ କ୍ୟାନ୍ସର ଇତିହାସ	Yes / No	ହଁ / ନା
	Menstrual history	ମାସିକ ଧର୍ମ ଇତିହାସ	Regular / Irregular / Others	ନିୟମିତ / ଅନିୟମିତ / ଅନ୍ୟାନ୍ୟ
	Education	ଶିକ୍ଷା ମାନ	Illiterate / Primary / Secondary / Higher secondary / Graduate & above	ନିରକ୍ଷର / ପ୍ରାଥମିକ / ମାଧ୍ୟମିକ / ଉଚ୍ଚ ମାଧ୍ୟମିକ / ସ୍ନାତକ ଓ ତାପରେ
	Occupation	ପେଶା	Homemaker / Laborer / Employed / Others	ଗୃହିଣୀ / ଶ୍ରମିକ / ନିଯୁକ୍ତ / ଅନ୍ୟାନ୍ୟ
	Socioeconomic status	ସାମାଜିକ–ଆର୍ଥିକ ସ୍ଥିତି	Modified BG Prasad scale (2024)	ସଂଶୋଧିତ BG ପ୍ରସାଦ ମାପଦଣ୍ଡ (2024)
II. Medical history	Existing comorbidities	ବର୍ତ୍ତମାନ ରୋଗ	Diabetes mellitus / Hypertension / Others	ମଧୁମେହ / ରକ୍ତଚାପ / ଅନ୍ୟାନ୍ୟ
	Sexual activity status	ଲୈଙ୍ଗିକ କ୍ରିୟାଶୀଳତା ସ୍ଥିତି	Yes / No	ହଁ / ନା
III. Knowledge and perception	Heard about cervical cancer	ସର୍ଭାଇକାଲ କ୍ୟାନ୍ସର ବିଷୟରେ ଶୁଣିଛନ୍ତି କି	Yes / No	ହଁ / ନା
	Source of information	ସୂଚନାର ଉତ୍ସ	Health worker / Television / Radio / Newspaper / Social media / Friends & relatives / Others	ସ୍ୱାସ୍ଥ୍ୟ କର୍ମୀ / ଟେଲିଭିଜନ / ରେଡିଓ / ସମ୍ବାଦପତ୍ର / ସୋସିଆଲ ମିଡିଆ / ମିତ୍ର ଓ ଆତ୍ମୀୟ / ଅନ୍ୟାନ୍ୟ
	Aware of affected age group	ପ୍ରଭାବିତ ବୟସ ଗୋଷ୍ଠୀ ବିଷୟରେ ଜାଣନ୍ତି କି	Yes / No	ହଁ / ନା
	Aware of cervical cancer screening program	ସର୍ଭାଇକାଲ କ୍ୟାନ୍ସର ସ୍କ୍ରିନିଂ କାର୍ଯ୍ୟକ୍ରମ ବିଷୟରେ ଜାଣନ୍ତି କି	Yes / No	ହଁ / ନା
	Knowledge of screening frequency	ସ୍କ୍ରିନିଂ କେତେ ବେଳେ କରାଯାଏ ଜାଣନ୍ତି କି	Yes / No	ହଁ / ନା
	Aware of screening locations	ସ୍କ୍ରିନିଂ କେଉଁଠାରେ ହୁଏ ଜାଣନ୍ତି କି	Yes / No	ହଁ / ନା
	HPV vaccination status	HPV ଟିକାକରଣ ସ୍ଥିତି	Yes / No	ହଁ / ନା
	Knowledge of signs and symptoms	ଲକ୍ଷଣ ଓ ଚିହ୍ନ ବିଷୟରେ ଜ୍ଞାନ	Multiple responses	ଏକାଧିକ ଉତ୍ତର
IV. Practices	Contraceptive practices	ଗର୍ଭନିରୋଧ ପ୍ରଥା	OCP / IUD / Hormonal / Permanent / None	ମୁଖେ ନିଆଯାଉଥିବା ଗୋଳି / IUD / ହରମୋନାଲ / ସ୍ଥାୟୀ / କିଛି ନୁହେଁ
	Menstrual hygiene practices	ମାସିକ ସ୍ୱଚ୍ଛତା ପ୍ରଥା	Sanitary pads / Cloth / Others	ସ୍ୟାନିଟାରୀ ପ୍ୟାଡ / କପଡ଼ା / ଅନ୍ୟାନ୍ୟ
	Ever undergone cervical cancer screening	କେବେ ସର୍ଭାଇକାଲ କ୍ୟାନ୍ସର ସ୍କ୍ରିନିଂ କରାଇଛନ୍ତି କି	Yes / No	ହଁ / ନା
	Type of screening procedure	ସ୍କ୍ରିନିଂ ପ୍ରକ୍ରିୟାର ପ୍ରକାର	VIA / Pap smear / Colposcopy / Don’t know	VIA / ପାପ୍ ସ୍ମିଅର / କଲ୍ପୋସ୍କୋପି / ଜାଣିନାହିଁ
	Encouragement for screening	ସ୍କ୍ରିନିଂ ପାଇଁ ପ୍ରୋତ୍ସାହନ	Self / Family members / Health personnel / Others	ନିଜେ / ପରିବାର ସଦସ୍ୟ / ସ୍ୱାସ୍ଥ୍ୟ କର୍ମୀ / ଅନ୍ୟାନ୍ୟ
	Advised by	କେଉଁଥିରେ ପରାମର୍ଶ ମିଳିଲା	ANM / CHO / Medical Officer / Others	ANM / CHO / ଚିକିତ୍ସା ଅଧିକାରୀ / ଅନ୍ୟାନ୍ୟ
V. Perceived barriers	Screening procedure is painful	ସ୍କ୍ରିନିଂ ପ୍ରକ୍ରିୟା ବେଦନାଦାୟକ ବୋଲି ଲାଗେ	Yes / No	ହଁ / ନା
	Lack of family support	ପରିବାରର ସମର୍ଥନ ଅଭାବ	Yes / No	ହଁ / ନା
	Difficulty accessing health facilities	ସ୍ୱାସ୍ଥ୍ୟ କେନ୍ଦ୍ର ପହଞ୍ଚିବାରେ ଅସୁବିଧା	Yes / No	ହଁ / ନା
	Personal embarrassment	ବ୍ୟକ୍ତିଗତ ଲଜ୍ଜା	Yes / No	ହଁ / ନା
	Fear of diagnosis	ରୋଗ ଚିହ୍ନଟ ହେବାର ଭୟ	Yes / No	ହଁ / ନା
	Belief disease develops after screening	ସ୍କ୍ରିନିଂ ପରେ ରୋଗ ହୁଏ ବୋଲି ବିଶ୍ୱାସ	Yes / No	ହଁ / ନା
	Mistrust in health centres	ସ୍ୱାସ୍ଥ୍ୟ କେନ୍ଦ୍ର ଉପରେ ଅଭିଶ୍ୱାସ	Yes / No	ହଁ / ନା
	Lack of awareness due to poor promotion	ଯଥେଷ୍ଟ ପ୍ରଚାର ନ ଥିବାରୁ ଜାଗୃକତାର ଅଭାବ	Yes / No	ହଁ / ନା
	Fear of stigma	କଳଙ୍କର ଭୟ	Yes / No	ହଁ / ନା
	Financial constraint	ଆର୍ଥିକ ସମସ୍ୟା	Yes / No	ହଁ / ନା
